# Taekwondo win-loss determining factors using data mining-based decision tree analysis: focusing on game analysis for evidence-based coaching

**DOI:** 10.1186/s13102-024-00906-5

**Published:** 2024-05-21

**Authors:** Minsoo Jeon, Hyosung Lim

**Affiliations:** 1 Department of International Sport, Dankook University, Chungcheongnam-do, Korea; 2 Department of Physical Education, Anyang University, Incheon, Korea

**Keywords:** Taekwondo, Win-loss determinants, Decision tree analysis, Evidence-based coaching, Martial arts, Female

## Abstract

**Background:**

Purpose In this study, the purpose of this study is to identify the determinants of winning and losing in taekwondo by applying decision tree analysis, one of the data mining techniques, based on the 2022 World taekwondo championships women’s competition.

**Methods:**

272 women’s games in the taekwondo championships in Guadalajara held by the WT in 2022 were used. For data processing, an independent sample t-test was performed for differences in game content variables according to the win/lose group, and a decision tree analysis was performed to confirm game content variables affecting the win/lose group. To check the predictive power of the model, classification accuracy, standard error, and misclassification estimates were calculated. All statistical significance levels were set at 0.05.

**Results:**

First, it was found that there was no statistically significant difference only in body attack (attempt) and number of kicking variables according to the winning and losing groups(*p* > .05), and there were differences in all other game content variables(*p* < .05). Second, as a result of conducting a decision tree analysis to confirm the determinants of winning and losing in taekwondo sparring, winning situation, tie situation, and number of kicks were identified as important variables.

**Conclusion:**

The World taekwondo championships are analyzed in the currently changed taekwondo competition rules to identify important factors, and at the same time, based on this, data-based coaching is expected to improve performance.

## Introduction

In the field of sports, various information is generated and provided based on quantitative game data [1]. A game information is released to the public for those watching the games and also for athletes and coaches to improve their performance. As the significance of game information increases within the realm of sports, so too does the interest in the field of sports analytics, which involves the collection and analysis of sports game data [2].

Among the information produced in sports, data on the factors determining win-loss outcomes is of particular interest. This information is utilized not only for athlete training and game tactics but also to provide engaging content for sports media and fans [3]. It plays a critical role in predicting the outcomes of sports games, and coaches use this data to make informed decisions. Therefore, information about the factors that determine win-loss in sports games is inevitably a critical element [3, 4].

In the area of sports analytics, efforts are made to explore and analyze internal game data to generate probability information that predicts game wins and losses or to identify game content variables that determine wins and losses [1]. These efforts are targeted at confirming the content related to winning and losing, which are the main interests in sports games. Since most participants in sports are inevitably interested in content related to winning and losing, many previous studies have continuously conducted research in this area.

Meanwhile, taekwondo, a sport that includes movements through kicks, fists, and steps, is characterized by high-intensity and low-intensity activities. In taekwondo [5, 6], research related to winning and losing has been reported [7], and considerable efforts are being made to enhance athlete performance [8, 9]. Additionally, time motion analysis [6] and notation analysis [10] of taekwondo athletes have been reported.

However, there is an aspect that we must pay attention to. Recent revisions to the rules of taekwondo competitions have introduced variables that affect athlete performance. The most critical new implementation is the transition to a ‘round-based win-loss system,’ where outcomes are determined round by round, and the athlete who first wins two rounds emerges victorious [11]. These changes affect not only game rules but also the tactics, techniques, and operations of the athletes, ultimately leading to differences in the factors that determine the win or loss of taekwondo games. According to the study by Oh et al. [12], the introduction of a round-based win-loss system has led to significant changes in score management and game operations, which are reported to have a direct correlation with match outcomes. In this context, recent rule changes such as the round-based win-loss system require a reevaluation of the existing variables used to predict victories. This highlights the urgent need for contemporary research to address these changes.

Despite the introduction of the round-based win-loss system, it is challenging to find studies conducted from an analytical perspective based on game data. The only research conducted post-introduction of this system has been to verify the awareness of changes in the game rules [12]. This study highlights the critical need for an updated analytical methodology that can accommodate the rapid changes in sports rules and athlete performance strategies, thereby responding to the growing interest in sports analytics.

Thus, the purpose of this study is to identify the determinants of winning and losing in taekwondo by applying one of the data mining techniques, decision tree analysis. Specifically, for the women’s division of the 2022 World taekwondo championships in Guadalajara, the difference between the winning and losing groups by game content variable was verified, and the game content variable that determines win-loss was confirmed through decision tree analysis. This result can be used as basic data for evidence-based coaching as well as to confirm the game operation information of the athlete changed through the game rule change.

## Methods

### Game data

In this study, the 2022 Guadalajara World taekwondo championships game video provided by the World taekwondo (WT) was selected as the target data for the game (https://www.youtube.com/@worldtaekwondo). Specifically, the women’s event that participated in the World taekwondo championships in Guadalajara, held by the WT in 2022, was selected as the competition target (Competition period: November 13–20, 2022). A total of 317 athletes participated in the women’s division, and a total of 272 games were collected (-46 kg: 35 games, -49 kg: 45 games, -53 kg: 36 games, -57 kg: 35 games, -62 kg: 39 games), -67 kg: 30 games, -73 kg: 30 games, + 73 kg: 22 games). In this study, to verify the difference in game content variables by winning and losing groups, it was divided into 4 weight classes (1 weight class: -46 kg, -49 kg); 2 weight classes: -53 kg, -57 kg; 3 weight classes: -62 kg, -67 kg; 4 weight classes: -73 kg, + 73 kg). If we were to analyze the results for all 8 weight classes, we could generate more detailed information. However, as we conducted the analysis based on a single World championships event, we encountered issues with the sample size. Therefore, to address this problem, we divided the data into Olympic weight classes (4 weight classes) through expert consultation and presented the results accordingly. However, in the decision tree analysis, it was calculated without classification due to the limitation on the number of cases. In this study, matches involving athlete injuries or forfeits were not included.

### Variable selection procedure

To achieve the purpose of this study, game analysis record variables were selected. As a variable selection procedure, first, previous studies on taekwondo competition analysis were explored. Specifically, the taekwondo game analysis variables (court management, attack type, and game situation variables) applied in the study of [13, 14], who performed the taekwondo game analysis, were primarily utilized. In addition, 3 taekwondo experts (taekwondo-related guidance for over 10 years, taekwondo national team member) and 2 physical education measurement evaluations were formed to collect opinions. As a result, expert opinion presented additional opinions on game posture, number of kicks, and attacking part (attempted, scored), and finally, a total of 7 variables were selected. The game record variables used in this study are shown in < Table 1>.

**Table 1:** Classification of recorded variables and variable levels

Variable	Classification of recorded variables		
Attack Area (attempt)	Trunk	Head	
Court Management	Central Court Operation	Court Occupancy Operation	Perimeter Court Operation
Attack Type	Pre-emptive Strike	Counterattack	
Attack Area (scored)	Trunk	Head	
Game Posture	Closed Posture	Open Posture	
Number of Kicks			
Game Situation	Wining	Losing	Tie

Central Court Operation: Conducting the game from the center of the court, similar to the opponent’s strategy

Court Occupancy Operation: A game management strategy that emphasizes controlling the court against the opponent

Perimeter Court Operation: Gameplay that involves less dominance over the court compared to the opponent

Pre-emptive Strike: Taking the initiative to attack before the opponent does

Counterattack: Responding to the opponent’s attacks with counterattacks for defense

Closed Posture: The stance where both athletes assume the same posture

Open Posture: The stance where both athletes assume different postures

### Research procedure and decision tree analysis

**Fig. 1:**
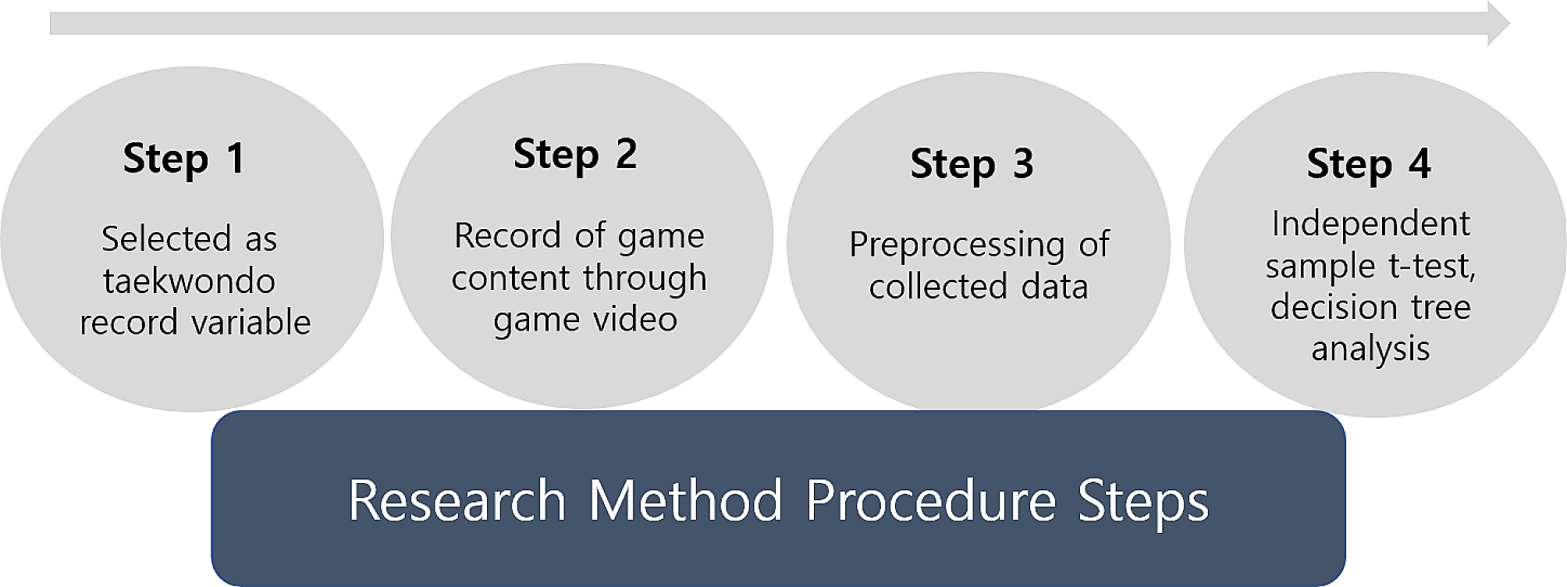
Research procedure steps

In this study, the research was broadly divided into four stages. First, selection of taekwondo game record variables, second, recording of record variables through game video, third, preprocessing of recorded data, and fourth, statistical analysis. The research procedure is shown in < Figure 1>. However, looking at previous studies that analyzed factors that determine win-loss in the field of sports, results are reported in various events such as basketball, handball, badminton, and water polo [15–17], methodology also uses logistic regression, discriminant analysis, decision tree analysis, etc. Loss determinants are derived and reported. Decision tree analysis, one of the methodologies applied in previous studies, is a statistical method that searches for decision-making rules based on multivariable and game data and models them into an optimal decision-making tree structure (1). Decision tree analysis is one of the most useful statistical methods for making predictions and classifications through collected data. In addition, it has the advantage of being easy to interpret the results and does not have to satisfy the basic assumptions of parametric statistics. It is used in a methodological way to determine the determinants of winning and losing in sports [3, 18–20].

### Notational analysis

In this study, the following procedures were followed for game records according to the selected variables. First, to record the game video in the form of a game record, it went through the process of quantifying the game content variable (court operation, attack type, first goal, etc.) selected in advance. The Excel 2013 program was used in the process of quantifying the game video in the form of a record. Second, the game record for the game video was recorded after 6 former taekwondo athlete, including the researcher, conducted education on the system and Excel program for the game record for about 2 weeks. The observer had more than 10 years of experience in taekwondo and was selected as a subject with a high level of awareness of the taekwondo game and proceeded with the recording. This is to minimize the error in the record while performing the game record, and after 2 weeks, to check the reliability between the observer, the reliability index ICC (intraclass correlation coefficient) was recorded as actual game data from 0.80 or higher (Minimum 0.824, maximum 0.945). For all variables except for the number of kicks and attack parts (attempts), only events in which points were scored in the game were recorded.

### Data processing method

The data processing method used to achieve the purpose of this study is as follows. First, an independent sample t-test was performed to verify the difference in game contents variables according to the women’s win/lose group. In addition, a decision tree analysis was performed to confirm the game content variables that affect the winning/losing group. In the decision tree analysis, the CHAID (chi-square automatic interaction detection) method, which is a method that can automatically find and analyze the interaction effect included in the analysis model, was applied. The independent variables used at this time were the 15 sub-variables (court occupation operation, central court operation, perimeter court operation, pre-emptive attack, counterattack attack, etc.) presented above (Table 1), and the dependent variable was selected as win-loss for each round. However, it is revealed that the variable for scoring was excluded because it has a very high correlation with win/loss. In the decision tree method, the maximum tree depth of 3 levels, the minimum number of cases, the parent node is 5, and the child node is 3. To do this, classification accuracy, standard error, and risk estimate were calculated and confirmed. The programs used for analysis were Excel 2013 (Microsoft corporation, Redmond, WA, USA) and SPSS 25.0 (SPSS science, Chicago, USA), and all statistical significance levels were set at 0.05.

## Results

### Analysis of game content variables according to win/lose group by weight class

In this study, a normality test was performed to perform parametric statistics, and in the normality test, all recorded variables were found to be *p* > .05 or higher, indicating that normality was satisfied.

#### Result of verifying the difference by variable in game content according to win-lose group in 1st and 2nd weight classes

In Table 2, an independent sample t-test was performed to verify the difference by variable in the game content according to the winning and losing groups in taekwondo women’s 1st weight class (-46 kg, -49 kg) and 2nd weight class (-53 kg, -57 kg).

**Table 2:** Results of differences verification by game content variables in the 1st and 2nd weight classes’ win-lose groups (using independent sample t-test)

Weight class	Variable classification		Winning group		Losing group		*t-test*		*Effect size(d)*
			Mean	SD	Mean	SD	*t*	*p*	
1st weight class	Attack Area (attempt)	Trunk Attack (attempted)	31.11	9.13	31.28	9.67	0.168	0.867	-0.018
		Head Attack (attempted)	5.57	3.74	4.83	3.74	1.880	0.061	0.198
	Court Management	Central Court Operation	1.81	1.60	1.01	1.25	5.289	< 0.001	0.555
		Court Occupancy Operation	0.90	1.05	0.47	0.82	4.279	< 0.001	-0.234
		Perimeter Court Operation	0.76	1.07	0.53	0.86	2.216	0.027	-0.438
	Attack Type	Pre-emptive Attack	2.04	1.52	1.03	1.27	6.799	< 0.001	0.443
		Counterattack	1.19	1.44	0.72	1.02	3.534	< 0.001	-0.283
	Attack Area (scored)	Trunk (scored)	1.98	1.59	1.20	1.38	4.963	< 0.001	0.522
		Head (scored)	1.24	1.14	0.55	0.90	6.361	< 0.001	0.755
	Game Posture	Closed Posture	0.66	0.92	0.36	0.73	3.338	0.001	0.360
		Open Posture	1.88	1.47	0.98	1.09	6.584	< 0.001	0.692
	Number of Kicks	Number of Kicks	36.01	14.9	35.35	15.03	0.418	0.676	0.060
	Game Situation	Winning	1.99	1.72	0.29	0.78	12.075	< 0.001	1.268
		Losing	0.57	0.96	1.30	1.44	5.642	< 0.001	-0.594
		Tie	0.90	0.60	0.43	0.57	7.574	< 0.001	-1.237
2nd weight class	Attack Area (attempt)	Trunk Attack (attempted)	29.44	6.81	30.50	7.86	1.309	0.192	-0.144
		Head Attack (attempted)	6.16	3.59	4.93	3.27	3.266	0.001	0.358
	Court Management	Central Court Operation	1.19	1.14	0.61	0.85	5.229	< 0.001	1.217
		Court Occupancy Operation	0.86	1.08	0.50	0.77	3.506	0.001	0.382
		Perimeter Court Operation	0.73	1.08	0.45	0.79	2.668	0.008	0.295
	Attack Type	Pre-emptive Attack	1.53	1.37	0.82	0.92	5.458	< 0.001	0.239
		Counterattack	1.05	1.30	0.56	0.82	4.123	< 0.001	0.447
	Attack Area (scored)	Trunk (scored)	1.45	1.31	0.97	1.07	3.657	< 0.001	0.399
		Head (scored)	1.12	1.17	0.40	0.67	6.787	< 0.001	0.501
	Game Posture	Closed Posture	0.67	1.07	0.39	0.68	2.813	0.005	0.311
		Open Posture	1.39	1.38	0.66	0.88	5.748	< 0.001	0.630
	Number of Kicks	Number of Kicks	37.94	8.19	37.86	8.29	0.087	0.931	0.089
	Game Situation	Winning	1.53	1.57	0.18	0.56	10.393	< 0.001	1.143
		Losing	0.47	0.85	0.99	1.17	4.663	< 0.001	-0.506
		Tie	0.78	0.61	0.37	0.56	6.225	< 0.001	-0.878

#### Result of verifying the difference by variable in game content according to win/lose group in 3rd weight classes and 4th weight classes

In Table 3, an independent sample t-test was performed to verify the difference by variables in the game content according to the winner and loser groups in 3rd weight classes (-62 kg, -67 kg) and 4th weight classes (-73 kg, + 73 kg) in the women’s taekwondo division. As a result, there was no statistically significant difference in body attack (attempted) (*p* = .839), head attack (attempted) (*p* = .401), and number of kicks (*p* = .548) in the third weight class. There were also statistically significant differences in all variables. In the 4th weight class, the body attack (attempted) (*p* = .441), head attack (attempted) (*p* = .293), closed posture (*p* = .205), and number of kicks (*p* = .882) were statistically significant. There was no significant difference, and statistically significant differences were found in all other variables.

**Table 3:** Results of difference verification by game content variable in the 3rd and 4th weight classes according to win-lose group (using independent sample t-test)

Weight class	Variable classification		Winning group		Losing group		*t*-test		*Effect size(d)*
			Mean	SD	Mean	SD	*t*	*p*	
3rd weight class	Attack Area (attempt)	Trunk Attack (attempted)	27.32	7.85	27.14	8.16	0.204	0.839	0.022
		Head Attack (attempted)	3.51	2.97	3.20	3.43	0.841	0.401	0.056
	Court Management	Central Court Operation	1.58	1.83	0.91	1.21	3.798	< 0.001	0.431
		Court Occupancy Operation	0.70	0.98	0.25	0.57	4.946	< 0.001	0.557
		Perimeter Court Operation	0.61	0.95	0.39	0.76	2.209	0.028	0.520
	Attack Type	Pre-emptive Attack	1.67	1.65	0.75	1.10	5.841	< 0.001	0.654
		Counterattack	1.04	1.18	0.59	1.06	3.593	< 0.001	0.399
	Attack Area (scored)	Trunk (scored)	1.80	1.82	0.84	1.17	5.570	< 0.001	0.293
		Head (scored)	0.90	1.04	0.47	0.87	3.923	< 0.001	0.448
	Game Posture	Closed Posture	0.56	0.90	0.30	0.67	2.817	0.005	0.326
		Open Posture	1.48	1.48	0.64	1.04	5.815	< 0.001	0.202
	Number of Kicks	Number of Kicks	32.99	8.53	32.39	9.23	0.601	0.548	0.067
	Game Situation	Winning	1.64	1.81	0.25	0.83	8.723	< 0.001	0.984
		Losing	0.49	1.10	0.88	1.20	-3.015	0.003	1.202
		Tie	0.75	0.54	0.42	0.61	5.136	< 0.001	0.569
4th weight class	Attack Area (attempt)	Trunk Attack (attempted)	27.01	7.15	27.76	7.86	-0.773	0.441	-0.100
		Head Attack (attempted)	4.78	3.61	4.31	3.22	1.055	0.293	-0.219
	Court Management	Central Court Operation	1.28	1.23	0.76	0.98	3.589	< 0.001	0.466
		Court Occupancy Operation	0.83	1.03	0.44	0.75	3.358	0.001	0.575
		Perimeter Court Operation	0.74	1.11	0.28	0.56	4.079	< 0.001	0.519
	Attack Type	Pre-emptive Attack	1.33	1.27	0.81	1.00	3.533	< 0.001	0.452
		Counterattack	1.29	1.35	0.53	0.85	5.192	< 0.001	0.460
	Attack Area (scored)	Trunk (scored)	1.69	1.43	0.96	1.17	4.347	< 0.001	0.559
		Head (scored)	0.73	1.00	0.22	0.48	4.988	< 0.001	0.646
	Game Posture	Closed Posture	0.49	1.00	0.35	0.69	1.271	0.205	0.591
		Open Posture	1.45	1.27	0.63	0.89	5.735	< 0.001	0.743
	Number of Kicks	Number of Kicks	34.92	8.10	35.08	8.41	-0.148	0.882	-0.019
	Game Situation	Winning	1.46	1.66	0.15	0.56	8.160	< 0.001	-0.129
		Losing	0.48	0.74	0.88	1.14	-3.201	0.002	-0.413
		Tie	0.81	0.62	0.42	0.52	5.241	< 0.001	0.673

### Determining factors of win-loss by game content using decision tree analysis

**Fig. 2:**
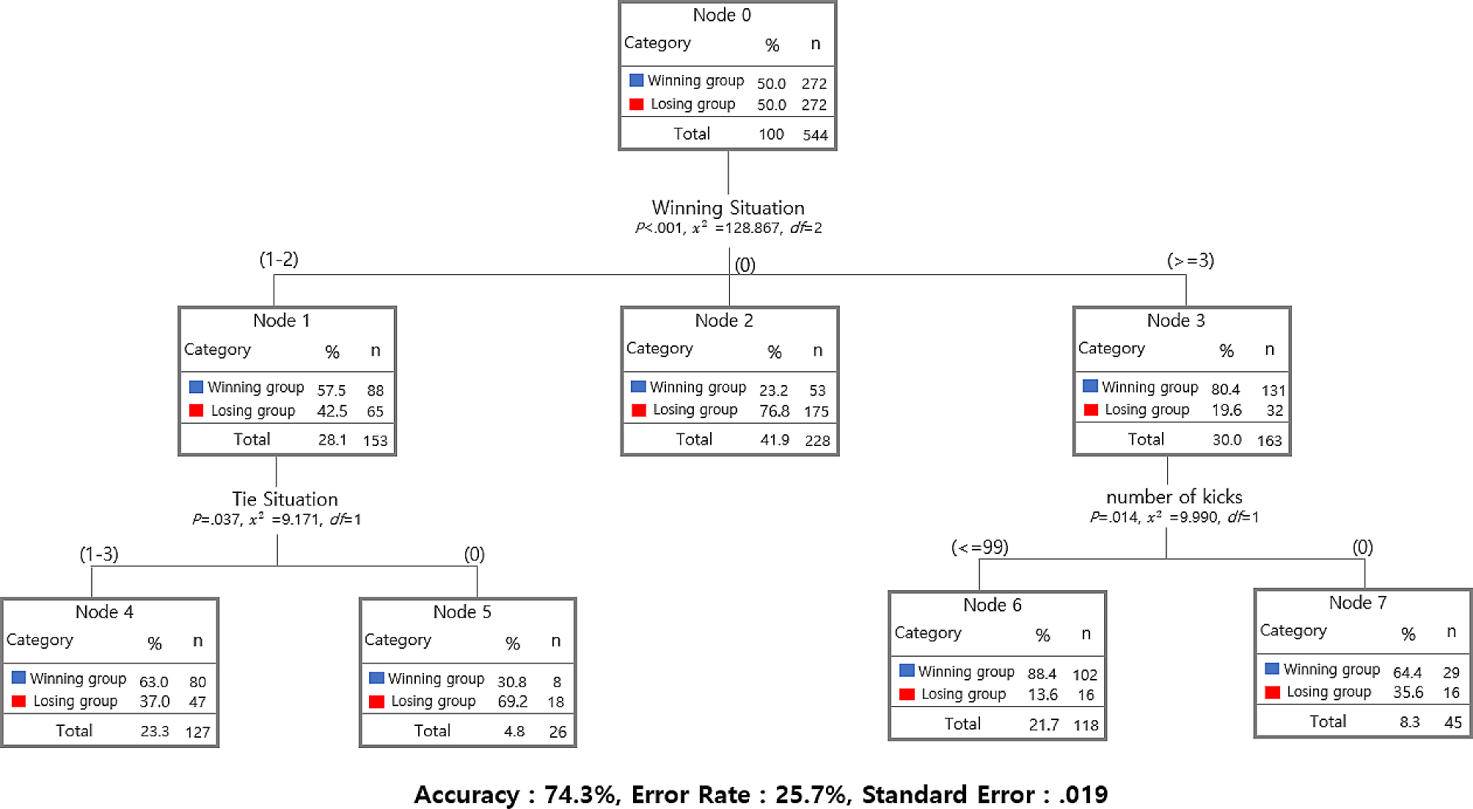
Results of analysis of win-loss determinants by game content using decision tree analysis

Figure 2 is the result of analyzing the determinants of winning and losing by content of taekwondo athlete using decision tree analysis. In the decision tree analysis, the dependent variable (win/lose variable) is located at the top, and each independent variable creates a branch to have a hierarchical structure. At this time, the independent variable located at the top is interpreted as a variable that is highly related to the dependent variable [17]. Looking at the results, the winning situation (x²=128.867, *p* < .001) was found to be the variable of game content that had the most important effect on the winning/losing group. In a winning situation, 80.4% of athlete scored 3 or more times, and 57.5% scored 1 - 2 times. On the other hand, it was found that 76.8% of 0 runs were lost. The probability of winning is 57.5% if you score 1 - 2 times in a winning situation, but it increases to 63.0% if you score 1 - 3 times in a tie situation.

In terms of the number of kicks, the probability of winning was the highest at 88.6% when more than 99 were performed, and the probability of winning was 64.4% even when the number of kicks was less than 99. < Table 4 > is a summary of the results of < Figure 2 > for each node variable. Node 3 (three or more points in a winning situation) corresponds to 163 (30.0%) cases out of a total of 544 cases, and 131 (80.4%) of them were found to be victorious. In node 4, 127 (23.3%) of the total cases were included, and 80 (63.0%) of them were found to be victorious. Node 6 contains 118 (21.7%) cases out of the total, of which 102 (86.4%) cases are analyzed to win. Classification accuracy for decision tree analysis: 74.3%, misclassification rate: 25.7%, standard error: 0.019.

**Table 4:** Summary of win-loss determinants by game content using decision tree analysis

Node	Winning group		Losing group		Prediction category	Game variable		
	*n*	%	*n*	%		Winning	Tie	Number of Kicks
1	88	57.5	65	42.5	Winning group	1, 2		
2	53	23.2	175	76.8	Losing group	0		
3	131	80.4	32	19.6	Winning group	>=3		
4	80	63.0	47	37.0	Winning group		1–3	
5	8	30.8	18	69.2	Losing group		0	
6	102	86.4	16	13.6	Winning group			<=99
7	29	64.4	16	35.6	Winning group			> 99

## Discussion

Among the information generated in sports, information on win-loss decision factors was of interest, which is used not only for athlete training and game tactics, but also to provide interesting information to sports media and fans [17]. This information plays an important role in predicting the outcome of sports games, and coaches use it to make various decisions. Therefore, information on the factors that determine win-loss in sports games is inevitably an important factor [17, 20].

In the field of taekwondo, research on game analysis related to win-loss have been actively conducted through various approaches, such as studies on win-loss and scoring techniques, attack areas, game operation, etc [4, 14]. . . However, it is true that most of the studies identify relationships based on one variable or are reported before the revision of the game rules, and there are limitations with the information that can be used in the field. Therefore, this study was conducted with the purpose of analyzing the determinants of winning and losing by applying decision tree analysis based on the 2022 held world championships.

First, as a result of confirming the difference in game performance variables according to the winning and losing groups by weight class, it was found that there was no statistically significant difference in the body attack (attempted) and number of kicking variables in all four weight classes. These results can be interpreted as a result reflecting the characteristics of electronic protectors and the changed game rules. Looking at recent taekwondo games, the number of kicks is relatively high, and it is progressing in the form of a game in which kicks are performed together when an opponent performs a kick. If kicking was performed to score points in the past, kicking is now performed for reasons such as preventing an opponent’s attack or not going out of bounds, so it can be interpreted that the frequency of kicking is similar regardless of performance [21]. Accordingly, in recent game rules, it was confirmed that the physical burden on athlete and the way the game is played are different from the past, and previous studies also reported that the change in physical strength management was the biggest due to the introduction of the rounds win-loss system. It can be interpreted in relation to the results [22].

Next, a decision tree analysis was performed to confirm the determinants of winning and losing in taekwondo sparring, and as a result, the winning situation variable was found to be the most important. Due to the nature of sports games, they are more likely to win when they are scoring, but from another aspect, the importance of the first score can be considered. In order to create a scoring situation in a taekwondo competition, first scoring should be performed as a top priority, and for this, tactics and training to acquire points first will be needed. Although the importance of the first goal has already been reported in many previous studies [21, 22], it is significant that it appeared as the most important factor even in the round and round-robin system where the current game rules have been changed. However, in order to win after scoring first, you will need to plan a strategy for the highest score of 3 to 5 points. This is a tactic reported in previous studies. For example, to score a 3-point head score, you must plan a strategy of attacking after getting close to the opponent or attempting to attack after attacking with a fist [23]. It is foreseeable. In particular, the advantage of decision tree analysis is that if you score more than 3 times in a winning situation, it provides information that will have an 80.4%-win rate. The second most important factors were the tied situation and the number of kicks. In the case of the number of kicks, there was no difference between the winning and losing groups, but the number of kicks was analyzed as a factor determining victory or defeat. The reason for this is judged to be because the round win or loss is decided within 2 min according to the introduction of the rounds win-loss system, so athlete perform many kicks in a short time without a search due to the reduced time compared to the past (6 min game time). Fundamental research on this emphasized cutting-edge exercise and anaerobic exercise to improve the performance of taekwondo athletes [6]. This is the result of considering the techniques and characteristics that occur in taekwondo. The results of this study also show that many kicks must be performed in a short period of time so that the feeling of kicking is important. In addition, it is interpreted that these results appeared because it has a system that has no choice but to try a lot of kicks due to the nature of the electronic body protector. In a study related to the frequency of kicks in taekwondo, it was suggested that it was impossible to conclude that the score was high even if the frequency of kicks was high [22]. As the result of an increase in the number of first strikes was reported, the results of this study were supported by suggesting a static relationship between the kicking frequency and the number of points scored.

Lastly, this study analyzed the determinants of victory and defeat based on the recently changed taekwondo competition rules and the World taekwondo championships. The reason why the women’s division was selected as the target in this study is because it requires a lot of time to be invested in analyzing taekwondo matches. Therefore, we plan to conduct research to analyze matches targeting the men’s division in the future. In this study, the research subject is limited to the women’s division, and the contents of the game were analyzed, and the limitations of the analysis were analyzed by performing the game record focusing on the event for the score in performing the game record. Nevertheless, this study is meaningful in that it analyzed recent game contents by applying decision-making analysis, one of the data mining techniques. Game analysis through various approaches provides coaches with a basis for effective coaching. This can complement their experience-based coaching and enable improved coaching through scientific evidence.

## Conclusion

The recent introduction of the round-based win-loss system in taekwondo has led to significant changes in the rules of the game, which have substantially influenced the performance and operation of matches. Such changes necessitate immediate sports analytics to provide athletes and coaches with the data needed to make informed decisions and adapt to these changes. However, despite the availability of surveys acknowledging the perception of the new system, there is a lack of substantial research analyzing the impact of these rule changes. Therefore, this study focuses on the 2022 World Taekwondo Championships in the women’s division, examining the differences in game content variables between winning and losing groups and applying decision tree analysis, a data mining technique, to identify the determinants of match outcomes. This approach is a well-validated analytical method previously used in sports research to determine factors influencing win-loss outcomes.

The major findings of this research are as follows: First, there were no statistically significant differences in torso attacks (attempts) and the number of kicks between the winning and losing groups, while other game content variables showed differences. Specifically, there were differences in the frequency of scoring from offensive moves, court management, type of attacks, game posture, and game situations depending on the performance. Secondly, the decision tree analysis conducted to identify the determinants of victory in taekwondo sparring revealed that the situation of victory, ties, and the number of kicks were significant variables. In a winning situation, scoring at least once classified an athlete into the winning group with an 80.4% probability, while in a tie situation, 63.0% were classified as winners. Additionally, with kick counts of 99 or more, 86.4% were categorized as winners.

This study has two significant implications: it makes an academic contribution to sports science and provides practical applications for coaches and athletes in the field. Firstly, just as the changes in rules have sparked alterations in athletes’ performance and strategic approaches, this study presents analytical results based on the recent modifications to the rules of taekwondo, thereby expected to fill a significant research gap that has not been addressed in other studies. Secondly, the study assists in immediately addressing changes, such as rule modifications in the field through academic support, enhancing the connection between sports practice and academia. It contextualizes the research within the broader field of sports analytics and underscores its unique contribution to understanding the impacts of rule changes in taekwondo. The study goes beyond merely identifying critical factors in the analysis of the World Taekwondo Championships under the revised rules and is anticipated to aid coaches in developing future training strategies and operations. These can be diversely utilized as foundational data for enhancing game performance, predictive modeling, data-driven training, and developing personalized programs.

## Data Availability

In terms of data availability, we will disclose data in accordance with BMC policy.

## Abbreviations

CHAID: Chi-square automatic interaction detection
WT: World taekwondo
ICC: Intraclass correlation coefficient
